# Emerging Concepts in Immune Thrombotic Thrombocytopenic Purpura

**DOI:** 10.3389/fimmu.2021.757192

**Published:** 2021-11-11

**Authors:** Aicha Laghmouchi, Nuno A. G. Graça, Jan Voorberg

**Affiliations:** Department of Molecular Hematology, Sanquin-Academic Medical Center Landsteiner Laboratory, Amsterdam, Netherlands

**Keywords:** TTP (thrombotic thrombocytopenic purpura), DRB1*11, DRB1*08, infection-immunology, microbiota, tolerance, autoimmune disease

## Abstract

Immune thrombotic thrombocytopenic purpura (iTTP) is an autoimmune disorder of which the etiology is not fully understood. Autoantibodies targeting ADAMTS13 in iTTP patients have extensively been studied, the immunological mechanisms leading to the breach of tolerance remain to be uncovered. This review addresses the current knowledge on genetic factors associated with the development of iTTP and the interplay between the patient’s immune system and environmental factors in the induction of autoimmunity against ADAMTS13. HLA-DRB1*11 has been identified as a risk factor for iTTP in the Caucasian population. Interestingly, HLA-DRB1*08:03 was recently identified as a risk factor in the Japanese population. Combined *in vitro* and in silico MHC class II peptide presentation approaches suggest that an ADAMTS13-derived peptide may bind to both HLA-DRB1*11 and HLA-DRB1*08:03 through different anchor-residues. It is apparent that iTTP is associated with the presence of infectious microorganisms, viruses being the most widely associated with development of iTTP. Infections may potentially lead to loss of tolerance resulting in the shift from immune homeostasis to autoimmunity. In the model we propose in this review, infections disrupt the epithelial barriers in the gut or lung, promoting exposure of antigen presenting cells in the mucosa-associated lymphoid tissue to the microorganisms. This may result in breach of tolerance through the presentation of microorganism-derived peptides that are homologous to ADAMTS13 on risk alleles for iTTP.

## Introduction

Thrombotic thrombocytopenic purpura (TTP) is a rare and severe life-threatening autoimmune disorder which is mostly acquired by adult patients with an annual incidence of approximately 4 cases per million people (1). The patients suffer from systemic clumping of platelets in different organs due to the lack of activity of the metalloprotease ADAMTS13 (a disintegrin and metalloprotease with a thrombospondin type 1 motif, member 13) (1–3). The metalloprotease ADAMTS13 is responsible for the rapid processing of newly released von Willebrand factor (VWF) (4, 5). VWF is synthesized in endothelial cells and upon stimulation ultra-large VWF (UL-VWF) polymers are released in the systemic circulation. UL-VWF unfolds under high shear stress and stretches into string-like structures which enables ADAMTS13 to cleave the unfolded UL-VWF polymers thereby preventing spontaneous thrombi formation in the microvasculature (6, 7).

The diagnosis of immune TTP (iTTP) is not only based on the deficiency of ADAMTS13 activity but also on the presence of autoantibodies targeting ADAMTS13 (8–13). It was found that the majority of TTP patients develop autoantibodies that bind to the spacer domain of ADAMTS13, which is crucial for binding and subsequent processing of VWF (14–18). Autoantibodies targeting N-terminal domains neutralize the proteolytic activity of ADAMTS13 while autoantibodies directed against other domains can cause accelerated clearance of ADAMTS13 from the circulation both resulting in the accumulation of UL-VWF polymers (10, 19, 20). The retained UL-VWF strings on the surface of endothelial cells cause the formation of platelet clots within arteries and arterioles leading to microvascular thrombosis that subsequently gives rise to hemolytic anemia (disruption of red blood cells) and thrombocytopenia (systemic low level of platelets) that may cause skin hemorrhages, presenting as purple-colored spots on the skin (purpura) (1, 9, 21).

The pathology of iTTP has been extensively studied for decades; the etiology of iTTP however, remains elusive. Here we will discuss our current knowledge on processes implicated with the development of autoimmune disorders (ADs) and their relevance for the development of autoimmunity against ADAMTS13, considering both genetic and environmental factors. In this review, we will also introduce a novel model describing the role of infections and microbiota in the onset and/or relapses of iTTP.

## HLA Associations in iTTP

The first suspicions concerning the involvement of genetic factors in TTP development rose from case reports describing the diagnosis of TTP in siblings (22, 23) and family members of different or same generation (mostly women) (24–27). Involvement of HLA was first suggested by the development of TTP in two HLA-identical brothers in the early 1980’s (25). The first large cohort study of HLA association in TTP was reported in the mid 1990’s by Joseph and co-workers (28). This cohort study elegantly showed the absence of the HLA class II HLA-DRB1*04 allele (encoded by the *DRB4* gene), indicating it to be a protective allele for TTP (28).

**Figure 1:**
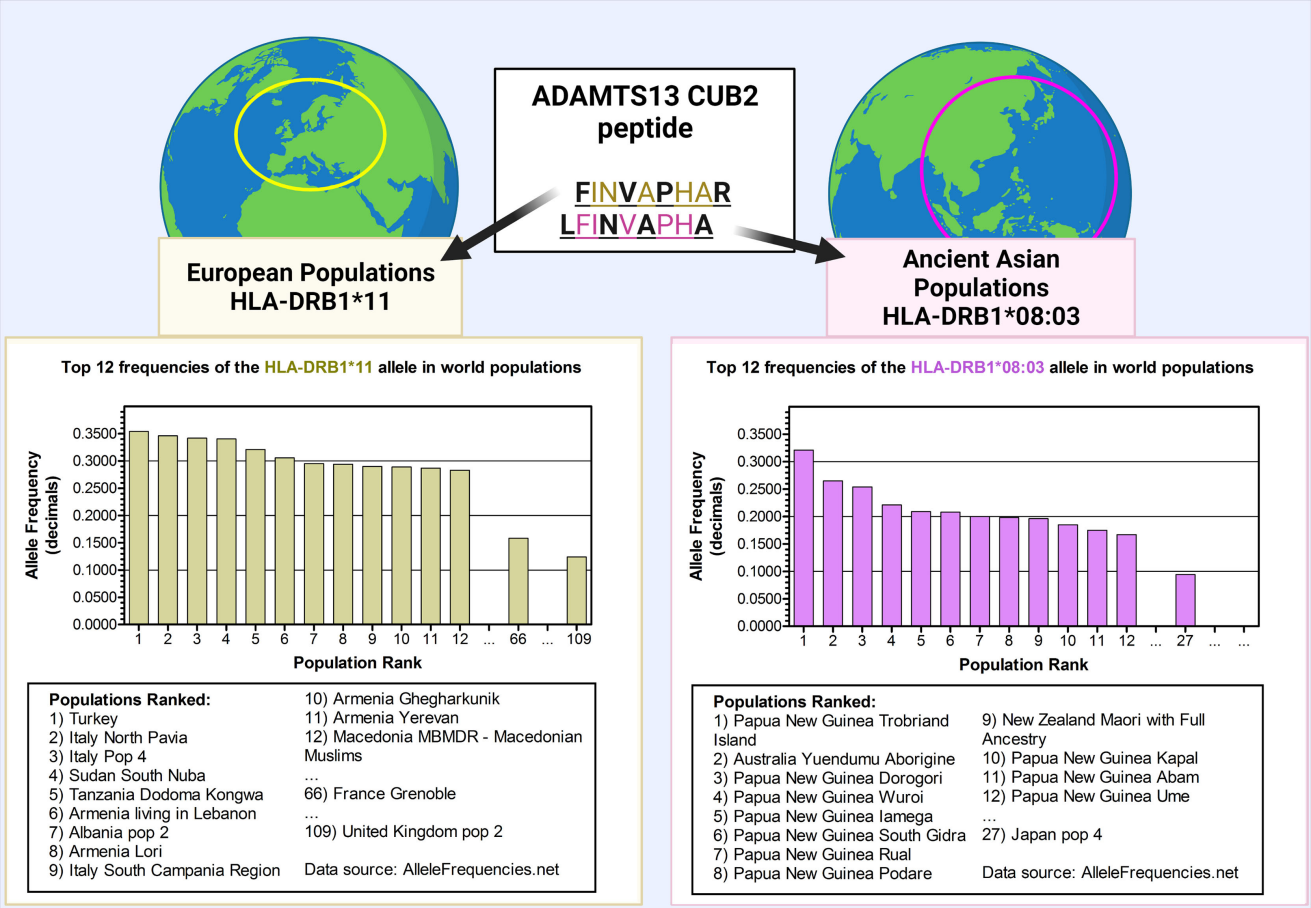
The frequency of the predisposing alleles HLA-DRB1*11 and HLA-DRB1*08:03 in world populations. The HLA-DRB1*11 allele was found to be a predisposing factor in the European Caucasian populations. However, in Asian populations, the predisposing allele was found to be HLA–DRB1*08:03. These alleles bind the same ADAMTS13 peptide derived from the CUB2 domain, however it is predicted that in the context of HLA-DRB1*08:03, the HLA-DRB1*11:01-presented peptide FINVAPHAR is shifted with one amino acid, becoming LFINVAPHA. The top 12 world populations are shown with the highest frequencies for each allele. The first countries in the list are the countries in their continent (France and UK for Europe, and Japan for Asia) where the main association studies were performed. HLA-DRB1*11 allele was found to be not frequent in far-east Asian populations. The first far-east Asian population in the list is the Taiwan Hakka pop 2 (rank number 184, with frequency of 0.0960; – other middle-eastern populations in Armenia, Georgia, Iran, Iraq, and central Russia, as well as central-Asian populations in Pakistan and India, rank higher). Conversely, HLA-DRB1*08:03 was found to be not frequent in European populations, with the highest-ranking European population on the list being Portugal center (ranked as 106, with frequency 0.0200). Data source: allelefrequencies.net (access date: 20^th^ July 2021). Figure created with Biorender.com.

After the clear separation of TTP from hemolytic uremic syndrome (HUS) (29) and the distinction between congenital TTP and iTTP (30–32) the first risk alleles for iTTP were found almost simultaneously by two independent groups (33, 34). The HLA-DRB1*11:01 and HLA-DRB1*11:04 alleles were found in different European Caucasian populations as the most prominent risk factors among the HLA-class II alleles (34, 35). The different studies also confirmed the earlier discovered protective allele HLA-DR53 (allele DRB1*04) (33–35). In later studies similar observations were done and additional HLA associations were found, which have been summarized in **Table 1**. In a more recent study, it was found that the HLA risk alleles in the Japanese population were substantially different than for the European Caucasian populations. The main HLA-DRB1 allele identified as a risk factor for iTTP was found to be HLA-DRB1*08:03 (38). In contrast to HLA-DRB1*11, which is highly expressed in the European population, HLA-DRB1*08:03 is an allele unique to individuals with East Asian ancestry (**Figure 1**). Additionally, the absence of HLA-DR3, -DR4 and -DR5 haplogroups (DR3/4/5*blank) and the higher frequency of HLA-DQA1*01:03 and HLA-DQB1*06:01 were also associated with iTTP in the Japanese population. In contrast, the haplotype HLA-DRB1*15:01/DRB5*01:01 (known to be in strong linkage disequilibrium) was identified as a protective factor in the Japanese population (38).

**Table 1:** HLA associations reported for iTTP.

HLA allele	Effect	Ethnicity	Author
HLA-B18	Predisposing	Caucasian	Coppo et al. (34)
DRB1*11	Predisposing	Caucasian	Scully et al. (33)
		Caucasian	Coppo et al. (34)
		Caucasian	John et al. (35)
		Children (mostly Caucasian)	Joly et al. (36)
DRB3*	Predisposing	Caucasian	Scully et al. (33)
DQB1*02:02	Predisposing	Caucasian	John et al. (35)
DRB1*11–DQB1*03	Predisposing	Caucasian	Sinkovits et al. (37)
DRB1*15–DQB1*06	Predisposing	Caucasian	Sinkovits et al. (37)
DRB1*08:03 or DRB3/4/5(blank) or DQA1*01:03 or DQB1*06:01	Predisposing	Japanese	Sakai et al. (38)
DRB1*04	Protective	Caucasian	Scully et al. (33)
		Caucasian	Coppo et al. (34)
		Children (mostly Caucasian)	Joly et al. (36)
DRB1*07–DQB1*02	Protective	Caucasians	Sinkovits et al. (37)
DRB1*13–DQB1*06	Protective	Caucasians	Sinkovits et al. (37)
DRB1*15:01 or DRB5*01:01	Protective	Japanese	Sakai et al. (38)

HLA associations have been found for several other ADs, with some of the associated HLA alleles being risk factors for one disease, but protective for other diseases (e.g. HLA-DRB1*04 is protective for iTTP in the general European Caucasian population, but a risk factor for giant cell arteritis (GCA) and Rheumatoid Arthritis) (39). Not all people that possess risk alleles develop the corresponding AD, which emphasizes the requirement of both genetic and environmental factors to lower the threshold for autoimmunity (37). To understand the role of HLA-DRB1*11 and other HLA class II alleles in the pathogenesis of iTTP, it is crucial to perform studies that provide mechanistic insight into the role of HLA class II in the onset of disease.

## Other Molecular Checkpoints in iTTP

Other genetic factors like single-nucleotide polymorphisms (SNPs) were repeatedly implicated in several other autoimmune diseases, although the mechanism by which they influenced autoimmunity was not always clear (40). The analysis of functionally relevant SNPs in Toll-Like-Receptor-9 (TLR-9) revealed TLR-9 +2848G and TLR-9 +1174A genotypes to be more frequent in TTP patients (41). TLR-9 is an endosomal pattern recognition receptor that recognizes unmethylated-CpG-containing DNA motifs from infectious microorganisms and directs Interferon-alpha production in humans and autoantibody development in mice (42). The gene *protein tyrosine phosphatase nonreceptor type 22* (*PTPN22*) encodes lymphoid specific phosphatase (Lyp), a potent inhibitor of T-cell activation, and the SNP C1858T is a risk factor in certain autoimmune diseases (43). However, an analysis of the *PTPN22* C1858T SNP did not show any difference between TTP patients and controls (37). More recently, two SNPs were found to be associated with the development of iTTP: rs6903608 (44) and rs9884090 (45). While there is lack of functional data, *in silico* analyses of these SNPs revealed that rs6903608 may increase expression of the risk HLA molecules for iTTP (44), while rs9884090 is associated with reduced expression of protein O-glycosyltransferase 1 (POGLUT1), implying that post-translational modifications may shape the immune response towards ADAMTS13 (45).

Post translational modifications of antigens have long been recognized to play a role in certain autoimmune diseases (46). ADAMTS13 is an extensively glycosylated plasma protein, containing both O-glycans and N-glycans (47). It is known that alterations in glycosylation patterns may have impact on the immunogenicity of antigens (48). It is possible that in iTTP reduced O-glycosylation of serines by POGLUT1 leads to altered antigen presentation in HLA risk alleles or altered T-cell receptor (TCR) recognition, however, functional data to confirm this hypothesis is still required. It is also noteworthy that ADAMTS13 as an antigen is more extensively modified by citrullination in the context of sepsis and in the elderly suffering from underlying comorbidities (49). This raises the possibility that citrullination of ADAMTS13 is another contributing factor for the loss of tolerance towards ADAMTS13, leading to iTTP. Citrullination was earlier described as a fundamental process in driving the autoimmune processes in rheumatoid arthritis and other inflammatory conditions (50), and was found to be capable of altering the specificity of TCRs towards T-cell epitopes, even though it did not impact HLA binding (51). Citrullination through peptidyl-arginine deiminase 4 (PAD4) drives the formation of neutrophil extracellular traps (NETs), which are normally triggered by infectious agents and contribute towards thrombosis by several mechanisms, including oxidation of ADAMTS13 methionines and possibly citrullination of ADAMTS13 through PAD4 (52). Biomarkers for NETosis were found to be elevated in iTTP patients (53). The conformational change of ADAMTS13 towards an open-state was also found as hallmark of iTTP and it is promoted through the binding of autoantibodies of patients (54, 55). Interestingly, non-inhibitory anti-ADAMTS13 autoantibodies also exist in healthy (56) and obese individuals (57). Like the pathogenic autoantibodies in iTTP patients, these should also promote the opening of ADAMTS13. The removal of C-terminal-domain N-glycans in ADAMTS13 may induce this open conformation state as well (58). The modifications and the confirmational change of ADAMTS13 may result in the availability of different and cryptic epitopes which may in turn help to contribute to the onset of iTTP.

## ADAMTS13-Derived Peptides Presentation

**Figure 2:**
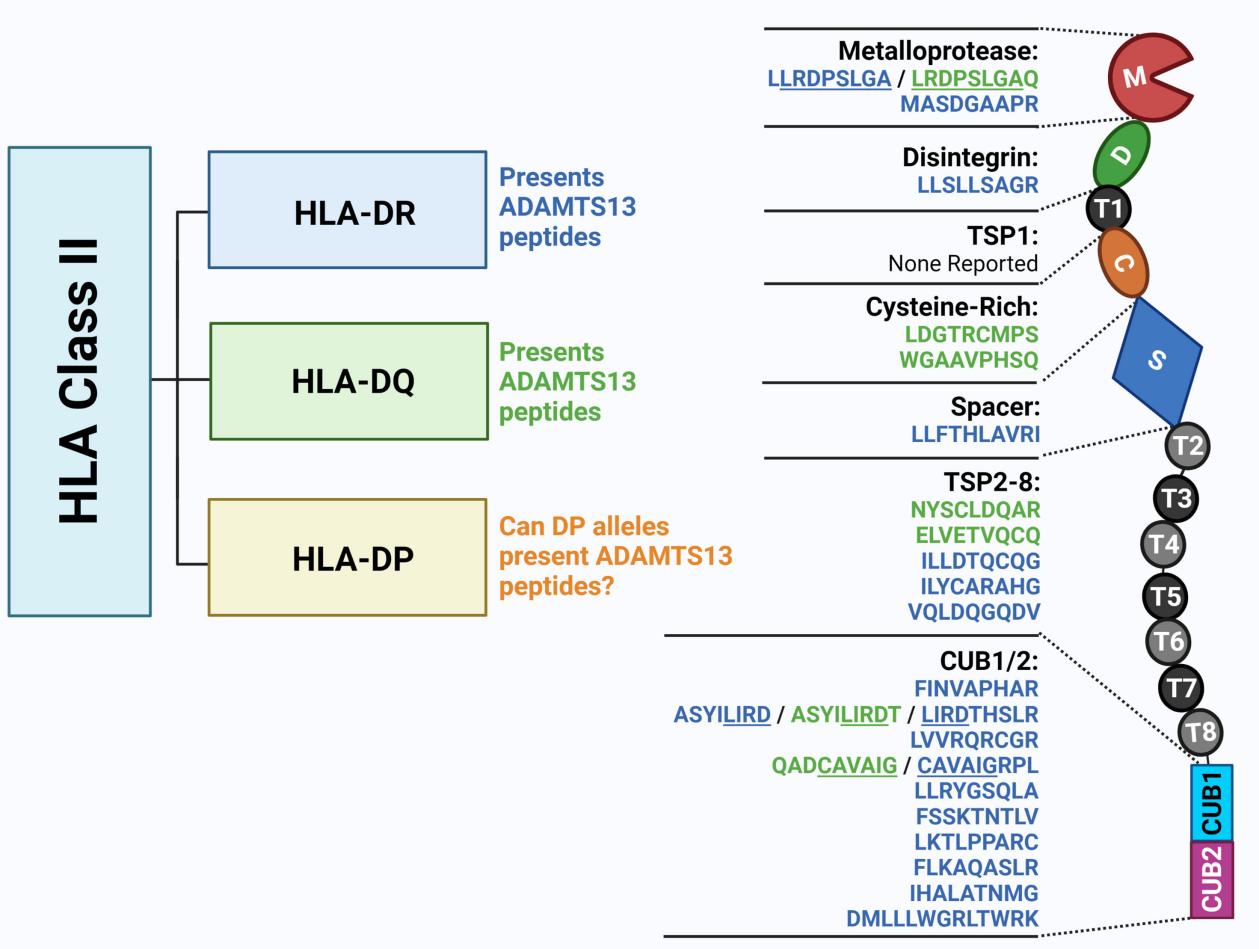
The ADAMTS13 peptidome presented by HLA Class II molecules. Previous studies unravelled ADAMTS13 peptides presented by HLA-DR and HLA-DQ molecules by peptide elution. The monocytes from HLA-DR and HLA-DQ-genotyped healthy donors were isolated and differentiated into dendritic cells (DC). DC were loaded with ADAMTS13, and upon lysis, HLA-II/peptide complexes were isolated by immunoprecipitation. Following elution ADAMTS13-peptides were identified through mass spectrometry. The 9-mer core sequences of the eluted peptide shown in this figure were identified through NetMHCIIpan 3.1 software (in blue for HLA-DR alleles and in green for HLA-DQ alleles) (63, 64). Figure created with Biorender.com.

The presentation of antigens in the context of HLA-class II molecules is required for the induction of adaptive immune responses. It is important to note that HLA-class II molecules usually bind ~12-20-mer peptides, while the core peptide inside the binding cleft normally consists of only 9-aminoacids, of which a few serve as anchor residues creating specific peptide binding motifs (usually involving aminoacid positions P1, P4, P6 and P9). HLA-class II peptides are not restricted by a closed peptide binding cleft structure of the HLA molecule as is the case for HLA-class I molecules (59). HLA-class II molecules display much more flexibility, allowing for differences in positioning of peptides within the peptide binding cleft and differences in the peptide flanking residues. Therefore, HLA-II molecules are known to have a certain degree of promiscuity - that is, one HLA-II molecule binds many different peptides. On the other hand, peptides have also been found to show a degree of promiscuity towards HLA-II molecules (60, 61). Consequently, some HLA-class II restricted T-cell receptors are capable of recognizing more than one HLA class II-peptide complex with different affinities (59, 60, 62). The ADAMTS13-peptidomes for HLA-DR and HLA-DQ (**Figure 2**) were dissected and it was revealed that the majority of eluents contained peptides derived from the ADAMTS13 CUB2 domain with the core sequence ‘FINVAPHAR’ (HLA-DRB1*11) or a different CUB2 peptide with the core sequence ‘LIRDTHSLR’. The latter was eluted from HLA-DRB1*03, an allele not formally associated with TTP (63). Peptides containing each of these core sequences were later shown to activate CD4 T-cells from TTP patients expressing the respective HLA-DRB1 alleles (65).

Elution experiments have demonstrated that promiscuity is a prominent feature of ADAMTS13 derived peptides. A notorious example is the ‘FINVAPHAR’ core peptide from the ADAMTS13 CUB2 domain which was shown to bind to different HLA-DR alleles (63). Interestingly, the core peptide ‘FINVAPHAR’ which was eluted from HLA-DRB1*11:01, was also predicted to be the highest affinity peptide for the risk factor HLA-DRB1*08:03 in the Japanese population, although the core sequence showed a shift of one amino acid, becoming ‘LFINVAPHA’ (**Figure 1**). This strongly suggests that HLA-binding peptides containing this core structure will likely be capable of binding to both HLA-DRB1*11:01 as well as HLA-DRB1*08:03 (38). Promiscuity was also shown for other ADAMTS13 peptides (e.g. the ADAMTS13-1239-1253 peptide, or GDMLLLWGRLTWRKM - a CUB1 peptide - and the core sequence ‘LLRDPSLGA’ - a metalloprotease peptide) (64). The ADAMTS13 peptidome for HLA-DQ was also assessed, and the same type of promiscuity was observed among these alleles. Peptides with the core sequence ‘QADCAVAIG’ (CUB1 domain) were eluted from two different haplotypes (64). Peptide promiscuity is also possible between different HLA class II molecules (i.e. the same peptide binding both HLA-DR and HLA-DQ) (66). The ‘QADCAVAIG’ core peptide found in the HLA-DQ elution study was also found for HLA-DR although the predicted core was slightly different (‘CAVAIGRPL’). The same applied to the peptides with the core ‘LLRDPSLGA’ eluted for HLA-DR, which were obtained also from HLA-DQ elution although with a slightly different predicted core sequence ‘LRDPSLGAQ’ (64). Overall, in the different elution experiments more peptides were eluted from HLA-DR alleles than HLA-DQ, which is probably influenced by the expression of the HLA molecules and the lower binding affinities of ADAMTS13 peptides in HLA-DQ (64) as was also shown for coagulation Factor VIII (66).

## The Role of Autoreactive CD4 T Cells in iTTP

The prominent HLA-class II association of iTTP combined with the presentation of ADAMTS13-derived peptides (including immunogenic peptides) on risk alleles for iTTP strongly supports a pivotal role for CD4 T cells in the immunopathogenesis of iTTP. Subclass and clonal analyses of anti-ADAMTS13 antibodies from iTTP patients provided evidence for isotype switching and affinity maturation (9, 10, 20, 67–69). These immunological processes further corroborate the role of CD4 T cells in the autoimmune responses against ADAMTS13. CD4 (helper) T cells are part of the adaptive immunity and are required for immune responses against pathogens or tumors. The T-cell receptors (TCR) of CD4 T cells bind to peptides in the context of HLA-class II (HLA-DR, HLA-DQ or HLA-DP) molecules on antigen-presenting cells (APC) (70, 71). The activation of CD4 T cells does not only depend on the binding of their TCR with peptide-HLA complexes but also on the binding of CD4 T cells with co-stimulatory (CD80 and/or CD86) and adhesion molecules (CD54) expressed by professional APC (72, 73). The naïve CD4 T cells differentiate upon activation by professional APC into various effector CD4 T-cell subsets, such as Th1 (74), Th2 (75), Th17 (76), T follicular (Tfh) (77) or regulatory T (Treg) cells (78). The CD4 T-cell fate is influenced by the cytokines produced by APC after which the differentiated T-cell subsets migrate to their target location to exert their function (73).

In ADs, auto-reactive CD4 T cells are used to induce the generation of autoantibodies by B cells. During T-cell development in the thymus, the T cells binding strongly to self-peptides presented in self-HLA are eliminated from the T-cell repertoire. However, a large part of the autoreactive T cells with a low or intermediate affinity escape negative selection and become part of the T-cell repertoire (71, 79–81). Autoreactive T cells involved in the development of iTTP and other ADs are required to possess a proper functional avidity to become activated by self-antigens. The avidity of T cells is determined by the TCR affinity to the peptide in the context of an HLA molecule. TCRs have been shown to bind differently to HLA-peptide complexes depending if the peptide is derived from a foreign antigen or from a self-protein. For foreign T-cell epitopes, the binding takes place with the TCR in a diagonal position relative to the HLA-peptide complex, with the CDR3 variable domains centered in the P5 position of the embedded peptide. In the case of self-peptides the docking may be altered, and examples where it may be shifted towards the N-terminal region of the peptide, or tilted in such a way that only the variable beta chain of CDR3 maintains contact with the HLA-peptide complex have been found (82). Besides the TCR binding, also the binding with stimulatory and adhesion molecules and the internal signaling transduction are important in the activation of CD4 T cells (83–85). In the context of iTTP, autoreactive CD4 T cells are hypothesized to be activated by APC that take up ADAMTS13 (using, for example, the macrophage mannose receptor (MR) or CD163) (86, 87). Upon endocytosis, ADAMTS13-derived peptides are loaded onto the HLA-class II molecules (63) and are recognized by the autoreactive CD4 T cells. Upon peptide recognition and co-stimulation, the CD4 T cells get activated and differentiate into effector CD4 T cells that secrete cytokines which subsequently stimulate antigen-specific B cells to differentiate into autoantibodies-producing plasma cells (20, 88).

The role of CD4 T cells in the development of iTTP is a clear given, as CD4 help is required in the production and affinity maturation of ADAMTS13-directed antibodies. However, the exact trigger for the activation of autoreactive CD4 T cells remains unclear. Like most ADs (86), iTTP is generally considered to be very rare (1), however the association between infection and the development of iTTP is evident. The high prevalence of infections at time of diagnosis (41%) (41) suggest that these pathogens are at least one of the triggers involved in the loss of tolerance towards ADAMTS13.

## Molecular Mimicry and Autoimmune Disorders

The etiology of ADs involves different factors including the genetic predisposition of the patient, but also the intricate interplay between the patient’s immune system and environmental factors (89, 90). The development of autoimmunity has often been associated with the occurrence of an infection which can be explained by the mechanism of ‘molecular mimicry’, the similarities between pathogen- and self-peptides resulting in the activation of autoreactive T or B cells in genetically susceptible individuals (89–92). Molecular mimicry is generally considered when four major criteria are met: 1) similarities between pathogen-derived antigens and self-antigens; 2) antibodies or T cells are detected that cross-react with both epitopes; 3) an epidemiological correlation/association between infection and onset of autoimmunity is present; 4) the development of the autoimmune disease is reproduced in an animal model by exposing the animal to the pathogen-derived cross-reactive antigen (89). In the past decades a variety of ADs were assumed to be caused by molecular mimicry as the antibodies or T cells demonstrated specificities directed against epitopes shared by pathogens and self-antigens. Only one autoimmune disease was found to fulfill all four listed criteria for molecular mimicry and that was Guillain-Barré syndrome resulting from *C. jejuni* infection (93). However, in many other ADs (type I diabetes, rheumatoid arthritis, systemic lupus erythematosus, Sjögren’s syndrome, systemic sclerosis, autoimmune thyroid disease, autoimmune liver diseases, autoimmune hepatitis, primary biliary cholangitis), and also in the context of vaccines, molecular mimicry was suggested to be the underlying mechanism but was not formally demonstrated due to lack of fulfillment of all the four criteria outlined above (89).

### Infections Associated With iTTP

**Figure 3:**
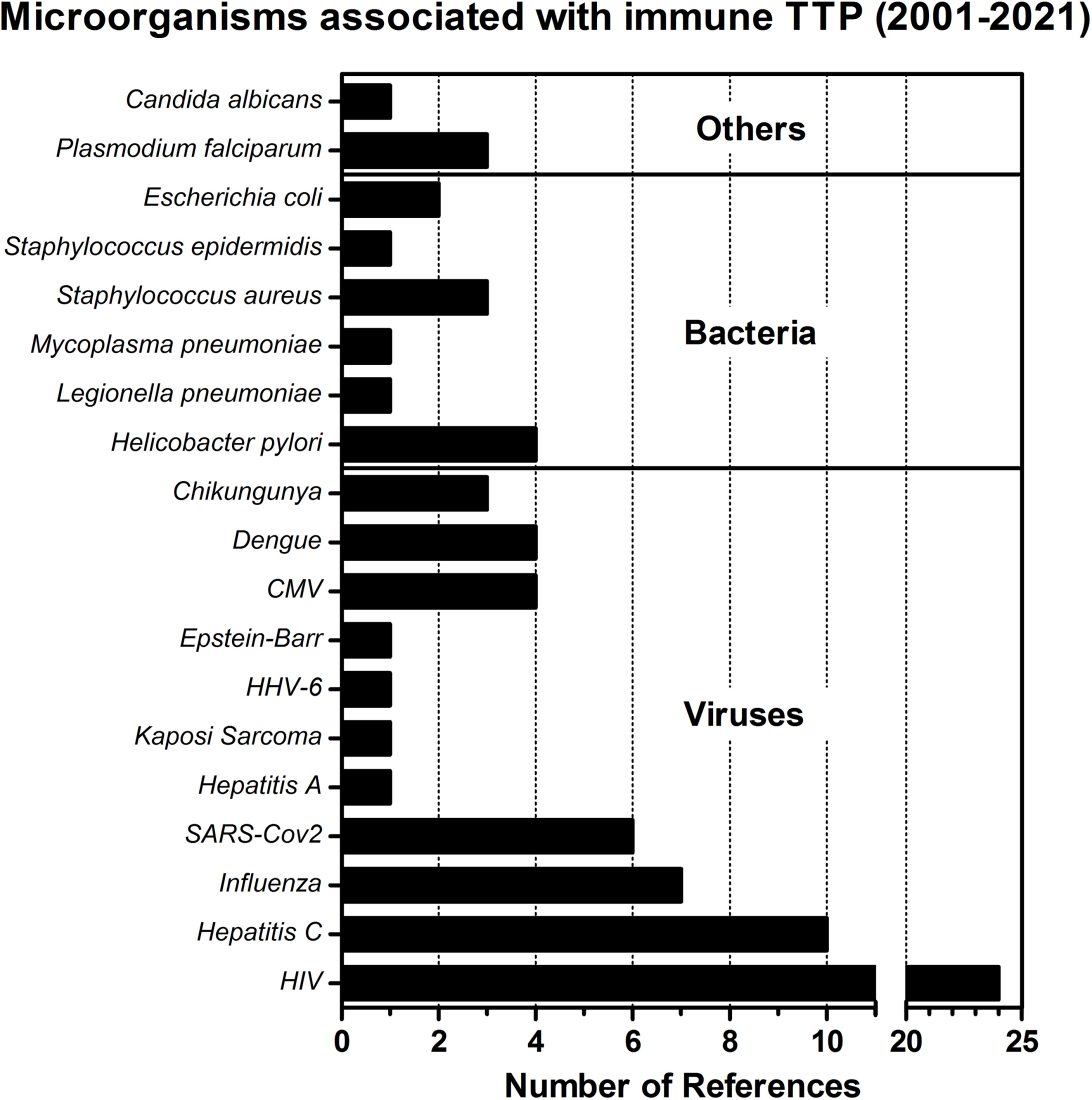
Microorganisms associated with immune TTP. The number of obtained references for different pathogens associated with the development of iTTP are presented in this bar-chart. The literature search was performed using the PubMed database, employing the search terms ‘thrombotic thrombocytopenic purpura’ AND ‘infection’ NOT ‘hereditary’ NOT ‘congenital’ NOT ‘immune thrombocytopenic purpura’ AND ‘Case Report’. References since 2001 (the year when ADAMTS13 was formally discovered and its structure annotated) were swept through to find infectious microorganisms that could be identified as a trigger for immune TTP in case reports under specific criteria: 1) the diagnosis of acquired TTP was done shortly after the infection was identified, or the infection was clearly associated as trigger of a relapse, 2) ADAMTS13 activity was preferably measured as <10%, 3) anti-ADAMTS13 autoantibodies were detected, confirming the diagnosis of immune TTP. The search was performed on the 28^th^ April 2021.

The development of iTTP has been associated with a variety of viral or bacterial infections as infections were found to be highly prevalent at time of diagnosis (41). We performed a literature search spanning the last 20 years which shows that several viruses, bacteria and other pathogens have been associated with the diagnosis of iTTP (**Figure 3**). The top three pathogens with a significant number of reports are: HIV, Hepatitis C, and Influenza (A) viruses, respectively. In the case of Influenza infection, it was previously observed that the heavy chain gene segment VH1-69 was preferred in antibodies targeting a highly conserved region in the hemagglutinin ectodomain of the virus (94). The same gene segment VH1-69 was also preferably used in antibodies directed towards ADAMTS13 (95–97). Future studies are required to address the possibility that the ADAMTS13 antibodies are in fact cross-reactive VH1-69 encoded antibodies targeting viral antigens (20). In **Table 2** an overview of all the microorganisms associated with the occurrence of iTTP is given. The pathogens’ target organs or target cells are listed, as well as one exemplary reference for each microorganism reporting a clear association between iTTP (defined as ADAMTS13 activity <10% and presence of anti-ADAMTS13 antibodies) and the infection. Associations between infection and iTTP must be made with caution. One must differentiate between secondary iTTP due to infection (that is, anti-ADAMTS13 autoantibodies developed due to the presence of the infectious microorganism) from secondary TTP caused either by the systemic inflammation and/or endothelial damage which commonly accompanies infections (13, 52, 115), or secondary TTP caused by drugs, or liver cirrhosis, or excessive alcohol intake (116, 117), as well as from other coagulation disorders well known to occur with infectious diseases (118, 119). The presence of autoantibodies together with ADAMTS13 activity < 10% is the defining factor for this differentiation.

**Table 2:** Pathogens associated with iTTP.

Pathogens		Target Organs/Cells	Reference
**Viruses**	Human Immune-deficiency virus (HIV)	T-lymphocytes	(98)
	Influenza A	Respiratory Tract	(99)
	SARS-CoV2	Respiratory Tract	(100)
	Hepatitis C and A viruses	Liver	(101, 102)
	Human gammaherpesvirus-8 (HHV-8, Kaposi Sarcoma)	Vascular Endothelium	(103)
	Human Herpes-virus type 6 (HHV-6)	T-lymphocytes, neurons, salivary glands	(104)
	Human gamma herpesvirus 4 (HHV-4, Epstein-Barr Virus)	B-lymphocytes, epithelial mucosa	(105)
	Cytomegalovirus	Epithelial mucosa	(106)
	Dengue	Langerhans cells in skin; vascular endothelium	(107)
	Chikungunya virus	Fibroblasts in skin, myocytes	(108)
**Bacteria**	*Helicobacter pylori*	Stomach	(109)
	*Legionella pneumoniae*	Respiratory Tract	(110)
	*Mycoplasma pneumoniae*	Respiratory Tract	(111)
	Atypical Community Acquired Pneumonia (undefined microorganism)	Respiratory Tract	(112)
	*Staphylococcus* spp.	Urinary tract	(113)
	*Escherichia coli*	Urinary Tract	(113)
	*Staphylococcus aureus*	Systemic (Septicaemia)	(113)
**Others**	*Candida albicans*	Systemic	(102)
	*Plasmodium falciparum* (Malaria/Blackwater Fever)	Liver; red blood cells	(114)

Furthermore, congenital TTP can be discovered because of infections and is frequently exacerbated as relapses or acute thromboembolic events due to infections (120, 121). The mechanisms involved in coagulation and thrombosis in infections are complex, transient, and ubiquitous to inflammatory cytokines (118, 119, 122). These also concur for reduced ADAMTS13 activity by tilting the balance in the ADAMTS13-VWF axis towards VWF in 3 ways: a) increase in VWF antigen levels (increased production and release of UL-VWF polymers from the endothelium); b) direct reduction of ADAMTS13 activity (123); and c) reduction of ADAMTS13 antigen (reduced ADAMTS13 synthesis in the liver and endothelium) (124). This is well documented in the case of COVID19 patients, where a “cytokine storm” is present and allows for such imbalance, with evidence for higher VWF antigen levels and lower ADAMTS13 activity in several cohorts (125). It is also evident through the accumulation of UL-VWF and lower ADAMTS13 activity in the plasma of COVID19 patients, which can be rescued by addition of recombinant human ADAMTS13 *in vitro* (126). Therefore, iTTP must formally be demonstrated in any association, and even if iTTP is proven, it is not always possible to attribute a direct causal relationship to one specific pathogen. For example, in an immunosuppressed patient multiple infectious agents may be present at the same time (notorious examples include HIV together with HCV (127), or opportunistic infections) and in other cases multiple conditions can be associated, such as other ADs or malignancies (128).

### Vaccines Associated With iTTP

Some reports described an association between vaccine administration and development of iTTP (examples listed in **Table 3**) (139). Influenza is commonly associated to TTP, and cases where ADAMTS13 autoantibodies were developed following influenza infection have been described (99). Globally, during the last 50 years only a few cases of TTP have been documented for influenza vaccines (**Table 3**) (139), even though 3100 million seasonal influenza vaccine doses have been administered in the United States of America alone since 1980 (140). A similar remark can be made of COVID vaccines, where, at the time of writing, only 2 cases of relapsing iTTP (134, 135) and 8 *de novo* iTTP cases (136–138, 141) were identified, among many millions of doses administered currently in the world. In this context, it is important not to confuse iTTP with vaccine-induced thrombotic thrombocytopenia (VITT), which has different mechanisms compared to iTTP (142, 143). Arguments for molecular mimicry in development of autoimmunity can still be made in the context of vaccines (144).

**Table 3:** Vaccines associated with iTTP.

Vaccines	References
Influenza Seasonal Vaccine	(129, 130)
Pneumococcal Vaccine	(131)
Influenza, Pneumococcal, Triple Diphtheria-Tetanus-Poliomyelitis Vaccines	(132)
Rabies Vaccine	(133)
COVID Vaccine	(134–138)

### Molecular Mimicry in iTTP

**Figure 4:**
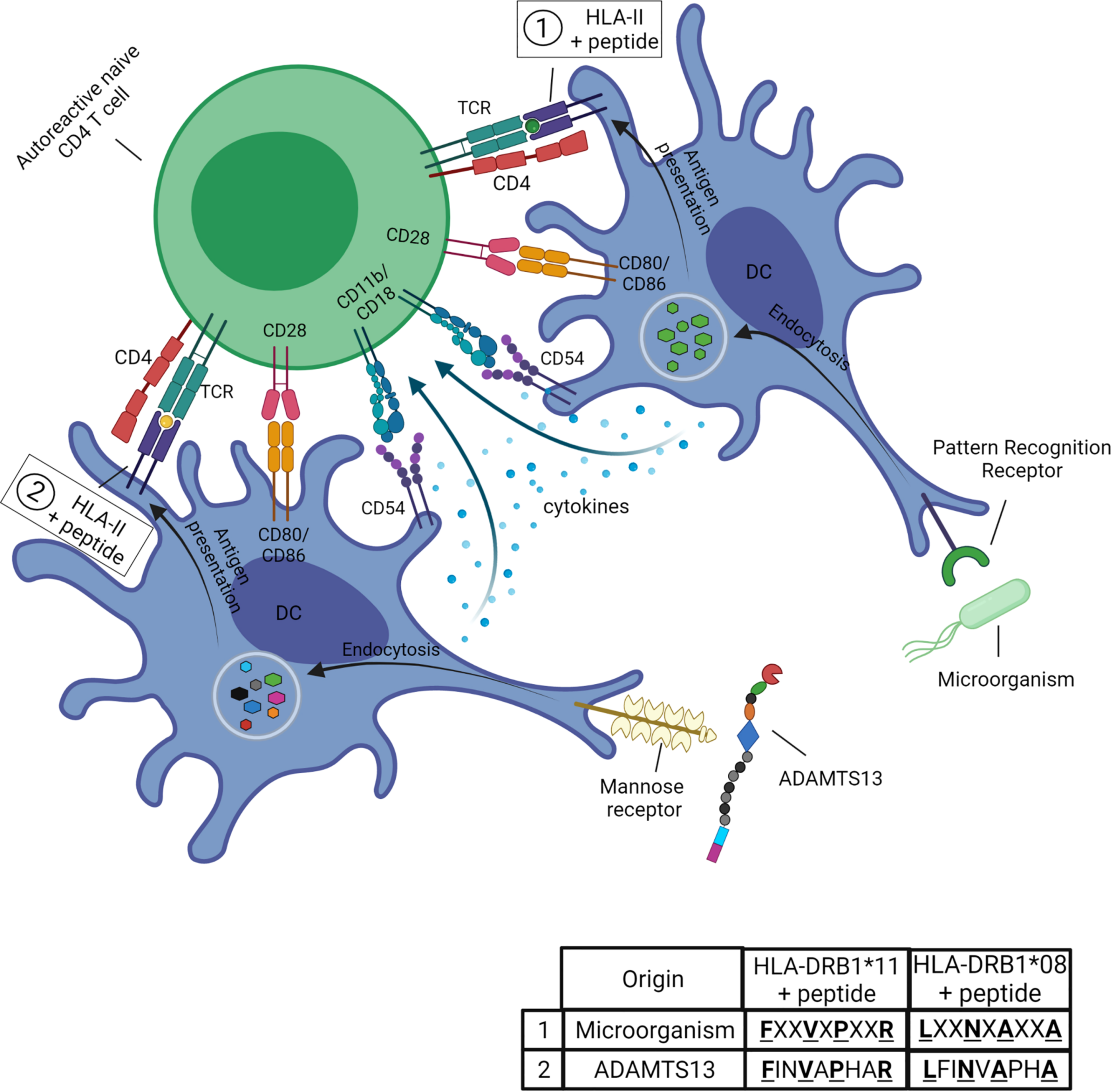
Potential role of molecular mimicry in iTTP. The microorganism is recognized by pattern recognition receptors (PRR) on APC (in this figure, dendritic cells, DC) followed by the uptake *via* the process of endocytosis. After antigen processing the microorganism-derived peptides (peptides 1 in table) are loaded onto the HLA-class II molecule (the risk allele HLA-DRB1*11 or HLA-DRB1*08:01, respectively) and presented to the TCR of an autoreactive CD4 T cell. The CD4 T cell cross-reacts with ADAMTS13-derived peptides (peptides 2 in table) presented in the HLA-II molecule (HLA-DRB1*11 or HLA-DRB1*08:01, respectively). The ADAMTS13-derived peptides show similarities with the microorganism-derived peptides for certain aminoacid positions (for example the anchor residues as indicated by the bold and underlined letters). The co-stimulatory and adhesion molecules (CD80/CD86 and CD54, respectively) on the APC are required for the activation of the autoreactive CD4 T cells. The ADAMTS13-derived peptides are obtained by the uptake of ADAMTS13 using the mannose receptor on APC. The cytokines produced by the APC are required for the differentiation of the naïve CD4 T cell into an effector CD4 T-cell population. Figure created with Biorender.com.

In the context of iTTP the mechanism of molecular mimicry (**Figure 4**) is hypothesized to occur when the microorganism is recognized by pattern recognition receptors (PRR) on APC followed by the uptake *via* the process of endocytosis. After antigen processing the microorganism-derived peptides (peptides 1 in table) are loaded onto the HLA-class II molecule (the risk allele HLA-DRB1*11 or HLA-DRB1*08:01, respectively) and presented to the TCR of an autoreactive CD4 T cell. The CD4 T cell cross-reacts with ADAMTS13-derived peptides (peptides 2 in table) presented in the HLA-II molecule (HLA-DRB1*11 or HLA-DRB1*08:01, respectively), these peptides show similarities with the microorganism-derived peptides for certain aminoacid positions. The co-stimulatory and adhesion molecules (CD80/CD86 and CD54, respectively) on the APC are required for the activation of the autoreactive CD4 T cells. The ADAMTS13-derived peptides are obtained by the uptake of ADAMTS13 using the mannose receptor on APC. The cytokines produced by the APC are required for the differentiation of the naïve CD4 T cell into an effector CD4 T-cell population (**Figure 4**).

The observed associations with either infections or vaccines in the development of iTTP have not been substantiated as of yet by a direct link to a specific pathogen antigen resembling ADAMTS13. The only exception is with Influenza, where a computational analysis was undertaken to assess if there are any sequences of the Influenza proteome that may resemble any part of the ADAMTS13 sequence and serve as cross-reactive T-cell epitopes (145). However, this analysis was done based on similarities at a 5-mer peptide level, less than the 9-mer core peptides necessary to bind the HLA molecules, and should therefore be refined. No cross-reactive B-cell epitopes have been formally identified yet. Overall, the evidence is suggestive that infections are linked to the onset and/or recurrence of iTTP. However, the mechanism by which these infections promote the development of iTTP is not yet clear.

## The Breakdown of Tolerance in Immune TTP

### Microbiota and the Development of Autoimmune Disease

The development of iTTP has been associated with both intrinsic as well as extrinsic factors. Genetic and other molecular checkpoints were found and suggested as predisposing intrinsic factors in patients. The association with different types of infection and the rarity of disease manifestation under the described conditions suggest that not only the genetic factors and the specific infection are involved, but that other environmental cues are at play. These should affect larger parts of the population and would tilt the balance from immune homeostasis to autoimmunity only in individuals with these predisposing factors, and only within specific situations that allow for the correct spatio-temporal alignment of the cross-reactive T and B cells.

One such environmental cue are the microbiota. Microbiota are known to be dependent on geographic regions, ethnicities and subsistence, with drastic variations among the different areas of the globe (146). The HLA system is known to interact with the gut microbiome, shaping its composition (147) and, simultaneously, the microbiome itself exerts systemic effects on the immune system, which has implications for immune homeostasis (148). Microbiota and dysbiosis have been the subject of many studies concerning the development of ADs, prominently in Type I diabetes mellitus (T1D). Initially, the increased incidence of T1D throughout time in genetically stable populations pointed towards effects coming from rapid improvements in public health and treatment of childhood illnesses that may have impacted the gut microbiota. Today there is a plethora of longitudinal prospective studies in humans and animal models supporting these hypotheses, including associations between HLA risk/protective alleles and microbiota composition and development of T1D (149–151).

The composition of microbiota was also found to influence the susceptibility to infections and the immune responses against infections (152). It has been suggested that in some ADs, the infections could cause the disruption of epithelial cell layers in organs (153). During immune homeostasis, the epithelial cell layer functions as a physical barrier to prevent the interaction of microbiota and antigen presenting cells in the mucosal associated lymphoid tissues to avoid undesirable activation of the host immune system. However, upon epithelial damage the microbiota can translocate beyond barrier surfaces like the gut, skin, lung and other mucosal surfaces and subsequently interact with immune cells (153). Microbiota-derived antigens - like pathogen-derived antigens - can share similarities with self-antigens which then may activate autoreactive T and B cells.

### A Role for Microbiota in Development of iTTP?

In the context of iTTP, there are no studies performed on the microbiota of patients to this date, and there are also no animal models that mimic the immune response against ADAMTS13 (154). However, additional ADs are frequently observed in iTTP patients, one of the most prevalent being Systemic Lupus Erythematosus (SLE). There are already extensive studies showing a role for microbiota in SLE development (155). This body of knowledge should be translational towards iTTP. The combination of infections and extensive exposure of the immune system to microbiota facilitates the “breach” in tolerance for self (see **Box 1**), and this may be the case for iTTP.

Box 1 **Self and non-self.**

The breach of self-tolerance towards ADAMTS13 is – as with any autoimmune process – dictated by factors of ‘self’ and ‘non-self’. Among the ‘self’ factors we have intrinsic factors like the possibility of HLA class I and class II peptides’ loading, which is directly dependent on the HLA haplotypes possessed by individuals, and dependent on possible variations in other ‘self’ antigens that may impact T-cell epitopes affinities to their respective HLA molecules (59), and other genetic factors that modulate the immune response (e.g. single nucleotide polymorphisms that modulate expression of proteins either directly involved in the immune synapse, or of other proteins involved in immune responses) (156–158), and – in a way, as an intrinsic factor – the microbiome, which is specific of each individual and region (146). On the other hand, the microbiome can also be considered as an extrinsic factor for ‘self’ (or an intrinsic factor for ‘non-self’), if we consider the human body as a superorganism – that is, a single entity composed of multiple organisms, in this case 1) the human body itself and 2) the total set of microorganisms that colonize the gut, vital to the shaping of immune responses that take place in the human body, and towards which the human immune system becomes inherently tolerant since birth (159). On the ‘non-self’ side, the most important extrinsic factor is the proteome of the total number of microorganisms the individual encounters throughout its life-span (regardless of their natural ability to cause diseases). A certain degree of overlap is inevitable between these types of factors due to the co-evolution of the human immune system shaped by the contact with microorganisms (160). On the edge of both types of ‘self’–’non-self’ factors stands the variable of time. Time influences the immune processes in two ways: 1) through the cumulative experience of host-microorganism interactions, allowing to build a B- and T-cell repertoire; 2) through a direct impact of aging on the individual’s characteristics at the levels of a) antigen repertoire and b) immune senescence (161–163). All these factors concur in this dynamic homeostatic process that dictates what is ‘self’, what is ‘non-self’, and when each of these entities are perceived as such by the human immune system (‘liquid-self’ theory) (161).

**Figure 5:**
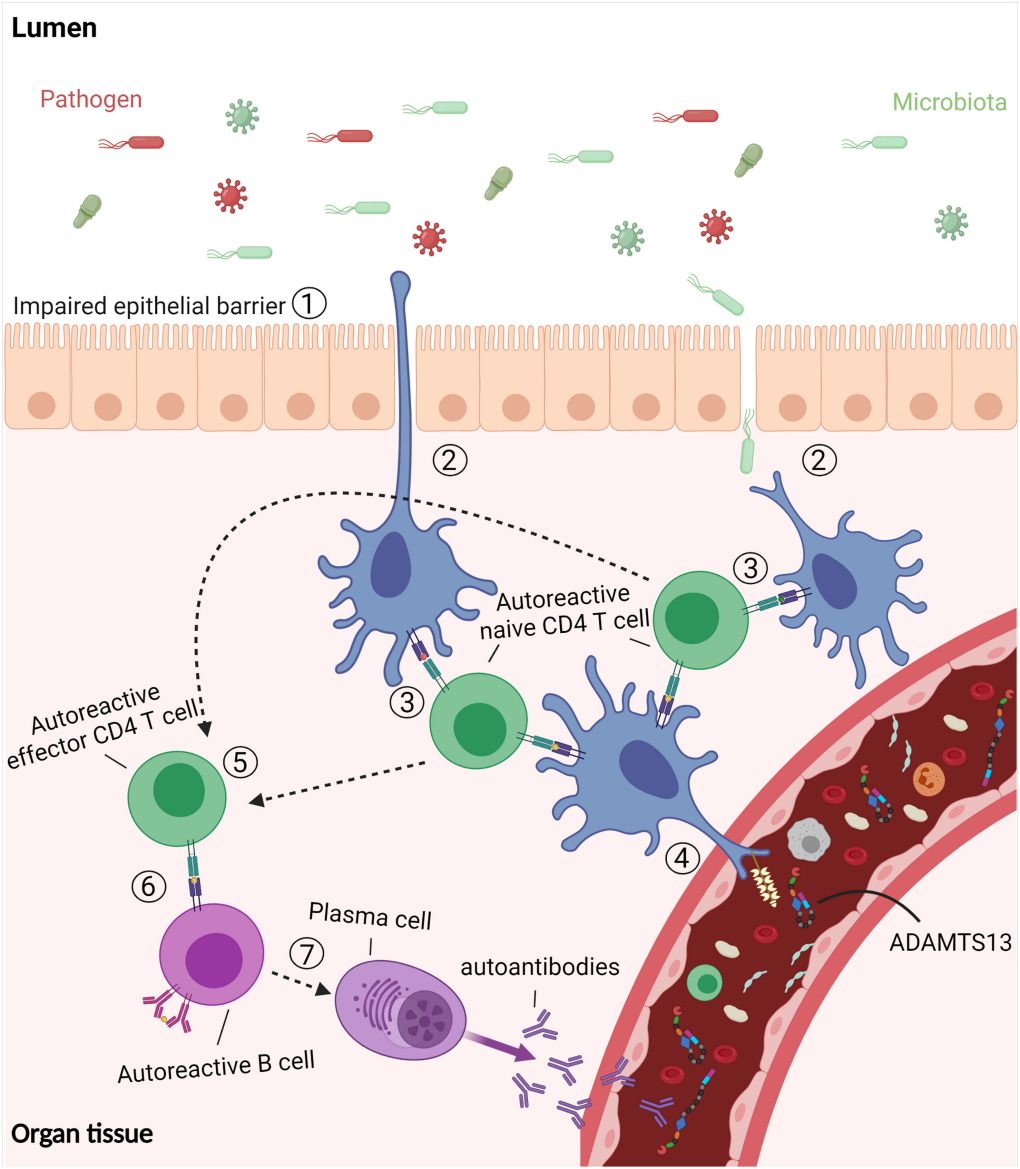
The “breach” of tolerance in immune TTP. Infections disrupt the epithelial cell layer which results in the exposure of the immune system to microbiota as well as pathogens. The dendritic cells (DC) acquire antigens from the microorganisms by phagocytosis. Following processing, the DC activate cross-reactive naïve CD4 T cells which also recognize antigens derived from ADAMTS13. The activated CD4 T cells differentiate into effector CD4 T cells which interact with autoreactive B cells to induce differentiation of the B cells into autoantibodies-producing plasma cells. Figure created with Biorender.com.

The current knowledge allows us to propose a model for the role of infections and microbiota in the development of autoimmunity against ADAMTS13 in iTTP patients (**Figure 5**). Infections are responsible for disrupting the epithelial cell layer, which results in the immune system being exposed to microbiota in addition to infectious pathogens (step 1). The APC (DC or macrophages) can acquire antigens from microorganisms as the epithelial cell layer is breached (step 2). The APC phagocytize and process the antigens, and present them to naive CD4 T cells (step 3). The autoreactive naive CD4 T cells that escaped the selection process cross-react against ADAMTS13-derived antigens presented after uptake and processing of ADAMTS13 protein (step 4). The activated CD4 T cells differentiate into effector CD4 T cells, which are involved in the induction of antibody responses (step 5). The autoreactive CD4 T cells interact with autoreactive B cells (step 6) to induce differentiation of the B cells into autoantibodies-producing plasma cells (step 7) (**Figure 5**).

## Concluding Remarks and Future Perspectives

The aim of this review was to unravel the etiology of iTTP by addressing both genetic and environmental factors involved in the induction of autoimmunity against ADAMTS13. Although the knowledge from other ADs gives insight to the possible immunopathogenesis of iTTP, several details of the processes leading to an immune response against ADAMTS13 remain to be uncovered. The findings in earlier studies seem very promising in pleading the case of molecular mimicry as a major underlying mechanism for the development of ADs and iTTP. Associations of HLA and iTTP have been identified, as they have been known for other ADs. ADAMTS13 peptides have been identified as T-cell epitopes in risk HLA alleles, and computational studies can be performed to find potential similar peptides from microorganisms, especially those commonly associated with iTTP at diagnosis. Some outcomes of such studies already exist. However, the search has to be continued and refined, both for infectious microorganisms and for microbiota, as done for other ADs (164). The use of bio-informatic tools in the homology search and the further validation using T-cell reactivity assays could substantiate the model as proposed in this review. More insight into the TCRs of iTTP patients is also needed in this field, and an animal model that properly reproduces the immune features seen in humans would provide a substantial advancement in the case of iTTP autoimmunity mechanisms. Additionally, cross-reactive B-cell epitopes remain to be formally identified, and research here may be aided by means of 3D-structure comparison software’s like CE-BLAST (165) or PDBeFold (166), by comparing available ADAMTS13 crystal structures with those of proteins from microorganisms. Future studies will further uncover the missing pieces of the puzzle that is autoimmunity and loss of tolerance towards ADAMTS13.

## Author Contributions

AL, NG, and JV wrote the manuscript and prepared the figures. All authors contributed to the article and approved the submitted version.

## Funding

Financial support was obtained from the Answering TTP foundation, Landsteiner Foundation for Blood Transfusion Research and The Netherlands Thrombosis Foundation. NG was supported by a grant from Sanquinnovate for the development of novel therapeutic compounds for treatment of iTTP. AL has been supported by a grant from the Answering TTP Foundation (grant year: 2018). NG has been supported by a grant from the European Union’s Horizon 2020 research and innovation program under the Marie Sklodowska-Curie grant agreement number 675746 (PROFILE) and a grant from Sanquinnovate (SQI00060).

## Conflict of Interest

NG and JV are inventors on a patent-application describing novel compounds for the treatment of immune TTP.

The remaining authors declare that the research was conducted in the absence of any commercial or financial relationships that could be construed as a potential conflict of interest.

## Publisher’s Note

All claims expressed in this article are solely those of the authors and do not necessarily represent those of their affiliated organizations, or those of the publisher, the editors and the reviewers. Any product that may be evaluated in this article, or claim that may be made by its manufacturer, is not guaranteed or endorsed by the publisher.
